# Construction and characterization of an infectious cDNA clone of coxsackievirus A 10

**DOI:** 10.1186/s12985-019-1201-1

**Published:** 2019-08-06

**Authors:** Qiliang Liu, Hanliang Dan, Xiaoping Zhao, Huoying Chen, Yongbei Chen, Ning Zhang, Zhijing Mo, Hongbo Liu

**Affiliations:** 1 Department of Laboratory Medicine, The Second Affiliated Hospital of Guilin Medical University, Guilin, Guangxi China; 2 College of Bio-technology, Guilin Medical University, Guilin, Guangxi China; 3 College of Laboratory Medicine, Guilin Medical University, Guilin, Guangxi China; 4 Center of Diabetic Systems Medicine, Guangxi Key Laboratory of Excellence, Guilin Medical University, Guilin, China

**Keywords:** Coxsackievirus A10, Infectious clone, ICR mouse, Mouse model

## Abstract

**Background:**

Coxsackievirus A10 (CA10) constitutes one of the four major pathogens causing hand, foot and mouth disease in infants. Infectious clones are of great importance for studying viral gene functions and pathogenic mechanism. However, there is no report on the construction of CA10 infectious clones.

**Methods:**

The whole genome of CA10 derived from a clinical isolate was amplified into two fragments and ligated into a linearized plasmid vector in one step by In-Fusion Cloning. The obtained CA10 cDNA clones and plasmids encoding T7 RNA polymerase were co-transfected into 293 T cells to rescue CA10 virus. The rescued virus was identified by SDS-PAGE, Western blotting and transmission electron microscopic. One-day-old ICR mice were intracerebrally inoculated with the CA10 virus and clinical symptoms were observed. Multiple tissues of moribund mice were harvested for analysis of pathogenic changes and viral distribution by using H&E staining, real-time PCR and immunohistochemical staining.

**Results:**

CA10 viruses were rescued from the constructed cDNA clone and reached a maximum titer of 10^8.125^TCID_50_/mL after one generation in RD cells. The virus exhibited similar physical and chemical properties to those of the parental virus. It also showed high virulence and the ability to induce death of neonatal ICR mice. Severe necrotizing myositis, intestinal villus interstitial edema and severe alveolar shrinkage were observed in infected mice. The viral antigen and the maximum amount of viral RNA were detected in limb skeletal muscles, which suggested that the limb skeletal muscles were the most likely site of viral replication.

**Conclusion:**

Infectious clones of CA10 were successfully constructed for the first time, which will facilitate the establishment of standardized neonatal mouse models infected with CA10 for the evaluation of vaccines and antiviral drugs, as well as preservation and sharing of model strains.

## Background

Hand, foot and mouth disease (HFMD) is typically a contagious childhood illness caused by human enterovirus (EV). Enterovirus A71 (EV71) and coxsackievirus A16 (CA16) are the primary pathogens associated with HFMD, while the proportion of HFMD caused by coxsackievirus A10 (CA10) has been increasing in recent years in different geographical areas(e.g., China [1–3], Finland [4], France [5], Thailand [6], Vietnam [7] and India [8]). In addition, CA10-associated HFMD commonly shows mild and self-limiting symptoms; nonetheless, a few cases present with various severe clinical manifestations, such as onychomadesis, herpangina, hyperCKemia, encephalitis, acute flaccid paralysis, neurorespiratory syndrome, and even death [5, 9–12]. Moreover, co-circulation of CA10 and other enteroviruses, such as EV71, CA16, and coxsackievirus A6 (CA6), increases the chance of genetic recombination and the emergence of new genetic variants of these viruses [3, 4, 13]. At present, with no effective vaccine and antiviral drugs, HFMD caused by CA10 has been a public health problem worldwide. Therefore, it is urgent to investigate infection mechanism and develop novel vaccines for the CA10 virus.

Infectious clones play an extremely substantial role in RNA virus research by reason that RNA genomes are difficult to preserve and manipulate. Besides, CA10 is a member of the enterovirus genus of the Picornaviridae family. Its genome is a positive single-stranded RNA with a length of approximately 7400 nucleotides, which has a single open reading frame (ORF) that is flanked by untranslated regions (UTRs) at the 5′ and 3′ ends. Research activities related to the viral pathogenesis, the functions of viral genes, viral infection and replication or vaccine development commonly involve genetic manipulations of enterovirus genome, which is nearly impossible to be conducted without an infectious cDNA clone of the viral genome. Infectious cDNA clones of several enteroviruses have been successfully constructed, including poliovirus, EV71 [14], CA16 [15], CA6 [16], CB3 [17], ECHO30 [18], ECHO25 [19], etc. However, there were no reports concerning CA10 infectious clones.

In the present study, an infectious clone from a strain of CA10 was established. The recovered CA10 virus had the same morphological and infectious characteristics with its parent strain. Moreover, it exhibited strong muscular tropism, induced multiple tissue damage and caused severe clinical symptoms or even death in neonatal mice. The construction of CA10 infectious clones may facilitate the next researches on CA10 pathogenic mechanism, vaccine development, etc.

## Methods

### Cell culture and virus

Human rhabdomyosarcoma (RD) cells were maintained in Dulbecco’s Modified Eagle Medium (DMEM; Gibco, Waltham, MA, USA) supplemented with 10% fetal bovine serum (FBS) (Gibco; Waltham, MA, USA), 100 U/mL penicillin, and 100 μg/mL streptomycin at 37 °C in presence of 5% CO_2_. The CVA10 strain, P148/ZS/CHN/2012 (No. MK645898), isolated from a clinical patient in Zhongshan area (Guangdong province, China), was propagated in RD cells and stored at − 80 °C in our laboratory.

### RNA extraction and reverse transcription polymerase chain reaction

CA10 was harvested in infected RD cellular supernatant after three freeze-thaw cycles and centrifugation. The viral RNA was extracted using QIAamp Viral RNA Mini Kit (Qiagen, Hilden, Germany), and then was reverse transcribed using PrimeScript™ II 1st Strand cDNA Synthesis Kit (Takara, Osaka, Japan), according to the manufacturer’s instructions. The synthesized first strand cDNA was used as a template for subsequent PCR amplification of CA10 genome fragments.

### Cloning of the full-length cDNA and T7 RNA polymerase DNA

**Fig. 1:**
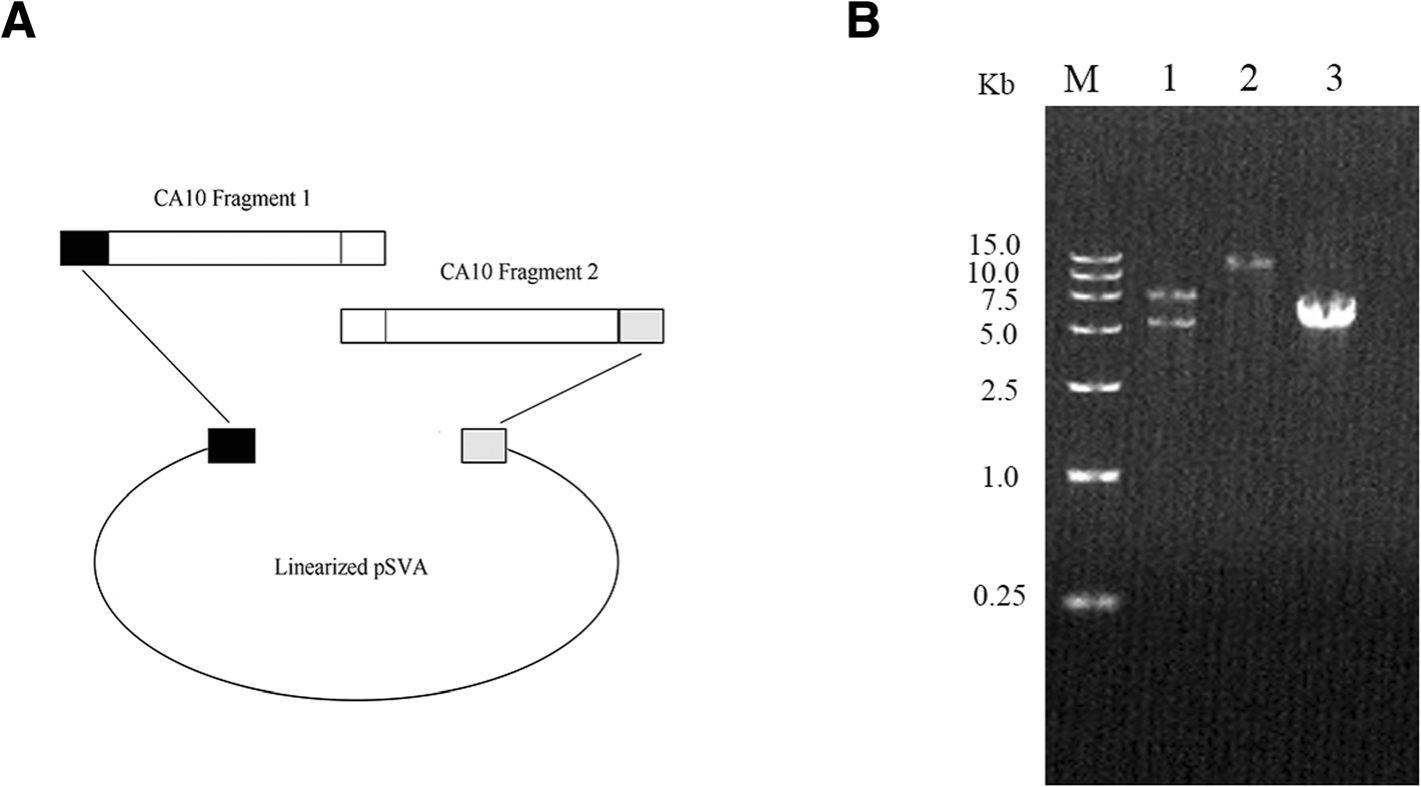
Construction of a full-length cDNA clone of CA10. **a** Schematic representation of construction of the full-length cDNA clone of CA10 by In-Fusion Cloning strategy. **b** Lane M is the DL15,000 DNA marker; Lanes 1 and 2 show the results of double (the band near 5.0 kb is the linearized pSVA vector and the band near the 7.5 kb is the CA10 DNA) and single enzyme digestion compared with the linearized pSVA vector for control (Lane 3)

The In-Fusion Coning strategy was used to construct the infectious clone of CA10, as shown in Fig. 1a. Primers were designed based on the CA10 genome sequence and the pSVA sequence. Primers (pSVA-R/CA10-F1, CA10-R1/CA10-F2, CA10-R2/pSVA-F) had an overlap of 20 bases (Table. 1). The pSVA vector, containing T7 promoter, was linearized by PCR using PrimeSTAR® GXL DNA Polymerase (Takara, Osaka, Japan) with pSVA-F/R primers. The genome of CA10 was amplified into two fragments with CA10-F1/R1 (for CA10 fragment-1) and CA10-F2/R2 (for CA10 fragment-2) primers. Then, the pSVA vector, CA10 fragment-1 and CA10 fragment-2 were assembled using In-Fusion® HD Cloning Kit (Takara, Osaka, Japan), resulting in plasmid pSVA-CA10. Then, the pSVA-CA10 clone was sequenced by Beijing Genomics Institute (Beijing, China). The T7 RNA polymerase gene was codon-optimized for mammalian cells, synthesized and then cloned into pLVX-Puro vectors by Beijing Genomics Institute.

**Table 1:** Primers used for the construction of CA10 infection clones

Primer name	Sequence (5’–3’)
pSVA-F	GTCGACAATTCTCATGTTTGACAGC
pSVA-R	CCTATAGTGAGTCGTATTACGCGGC
CA10-F1	GTAATACGACTCACTATAGGTTTAAAACAGCCTGTGGGTTGTACC
CA10-R1	CGCCGAGTCCCTTGATATAGTCAGACACTCCCTG
CA10-F2	CTATATCAAGGGACTCGGCGATGCTTTT
CA10-R2	CAAACATGAGAATTGTCGACTTTTTTTTTTTTTTTTTTTTTTTTTGCTATTC

### Co-transfection to rescue virus

To rescue the CA10 virus, 293 T cells seeded at density of 5 × 10^5^ cells / well into a 6-well plate were grown in Opti-MEM® I reduced serum medium (Gibco, Waltham, MA, USA) without antibiotics for 24 h. The co-transfection mixture, which contained 1 μg pSVA-CA10 DNA, 1.5 μg pLVX-Puro-T7 RNA polymerase DNA, 7.5 μL P3000™ Reagent, 10 μL Lipofectamine™ 3000 Reagent and 250 μL Opti-MEM medium per well was prepared according to Lipofectamine™ 3000 Reagent (Thermo Fisher Scientific, Waltham, MA, USA) protocol and inoculated into 293 T cells and incubated for 3 days at 37 °C. The rescued CA10 viruses were harvested by freeze-thawing. The supernatant was then inoculated to RD cells in serum-free DMEM medium. At 24 h post-inoculation, the cells displayed almost 80% cytopathic effect (CPE), suggesting that the co-transfection of pSVA-CA10 DNA with pLVX-Puro-T7 RNA polymerase DNA can produce infectious viruses.

### Virus titration

Virus titers were determined by microtitration using RD cells and expressed as the 50% tissue culture infectious dose (TCID_50_). Briefly, 96-well plates were seeded with a density of 1 × 10^4^ cells/ well in DMEM supplemented with 10% FBS and incubated at 37 °C for 24 h prior to infection with serial dilutions of the virus. Each dilution was inoculated with 8 wells for 200 μL per well. The plates were cultured for 8–10 days, and observed for CPE. The TCID_50_ values were calculated according to the Reed–Muench method [20].

### Purification of viruses and transmission electron microscopy (TEM)

To purify CA10 particles, the virus culture supernatant was harvested and the cell debris was removed by three times of centrifugation at 8000 rpm using a #3335 rotor (Thermo Fisher Scientific, Waltham, MA, USA) for 20 min each time. The virus sample was precipitated by incubation with 8% polyethylene glycol (PEG) 8,000 in 0.5 M NaCl at 4 °C for 12 h. The mixture was centrifuged using a 45Ti rotor (Beckman Coulter Inc., Brea, CA, USA) at 24,000 rpm for 30 min at 4 °C. The pellets were resuspended in phosphate-buffered saline (PBS) and further purified by a 10~50% continuous sucrose gradient centrifugation at 32,000 rpm in a SW60 rotor at 4 °C for 3 h. The fractions at 20~40% sucrose were collected and dialyzed against three exchanges of 500 mL PBS at pH 7.4, and then stored at 4 °C. The formation of CA10 particles was analyzed by negative staining electron microscopy according to a previously described method [21]. Briefly, purified viruses were adsorbed to 200 mesh carbon-coated copper grids and incubated for 10 min at room temperature. The grids were then washed once with PBS and stained for 45 s with 2% phosphotungstic acid. Specimens were evaluated using an electron microscope.

### SDS-PAGE and Western blotting

SDS-PAGE analysis of CA10 virus was performed in 10% SDS polyacrylamide gels according to the protocol as previously described [21]. For immunoblotting, CA10 viral proteins were directly electro-transferred onto the polyvinylidene difluoride (PVDF) membrane and probed with mouse anti-CA10 VP1 antibody, followed by a corresponding horseradish peroxidase (HRP)-conjugated secondary antibody. Membranes were developed by Pierce™ ECL Plus Western Blotting Substrate (Thermo Fisher Scientific, Waltham, MA, USA) and signals were recorded by a gel imaging system (Chemi-Doc XRS^+^; Bio-Rad Laboratories, Inc., Hercules, CA, USA).

### Animal experiments

Specific pathogen-free (SPF) Institute of Cancer Research (ICR) pregnant mice were purchased from Silaikejingda Laboratory Animal Co., Ltd., Hunan, China. All animal experiments were undertaken in accordance with protocols approved by the Institutional Animal Care and Use Committee and Ethics Committee of Guilin Medical University (Guilin, China). To evaluate the virulence of the rescued CA10, grouped one-day-old ICR mice were intracerebrally challenged with 10-fold serial dilution (10^7^~10^1^ TCID_50_) of passaged CA10 (20 μL CA10 sample per mouse). The control mice were injected with 20 μL of PBS via the same route and maintained in a separate cage from the infected mice. Every group contained 8 neonatal ICR mice, and all mice were monitored daily for body weight, clinical illness and death until 21 days post-infection. Clinical grading was carried out as previously reported [22]: 0, healthy; 1, lethargy and inactivity; 2, wasting; 3, limb-shake weakness; 4, hind-limb paralysis; and 5, moribund and death.

### Real-time PCR

Total RNAs were extracted using MiniBEST Universal RNA Extraction Kit (Takara, Osaka, Japan) from the same weight of tissue homogenates (brain, intestine, limb skeletal muscles, heart, liver, and lung), respectively, and then were reverse transcribed with PrimeScript™ RT reagent Kit (Takara, Osaka, Japan) according to the manufacturer’s instructions. For quantification, real-time PCR analysis was performed by using TB Green™ Fast qPCR Mix (Takara, Osaka, Japan) with primers (Forward, 5′-GGCGATCCTGTGGAGGATATAAT-3′, Reverse, 5′- TCTCTAATCGGTGTGAACTGGGA-3′) in Bio-Rad CFX96 system (Bio-Rad Laboratories, Inc., Hercules, CA, USA). Real-time PCR procedure was conducted as follows: for 15 min at 95 °C, followed by 40 cycles of 95 °C for 10 s, 60 °C for 20 s and 72 °C for 20 s. The quantified pSVA-CA10 plasmid was 10-fold serial diluted and used as standard sample for generating a standard curve. Virus loads were expressed as log10 copies/mg of tissues.

### Histopathological and immunohistochemical analysis

For histopathological and immunohistochemical analysis, each of one-day-old ICR mice infected with 20 μL of 10^4^ TCID_50_ CA10 in a moribund state or with PBS as control was euthanized. Brain, limb skeletal muscles, intestines, and lung were separately harvested and immediately fixed by 4% formalin/PBS for at least 3 h at room temperature. Fixed tissues were bisected, embedded in paraffin and sectioned on thickness of 4 mm. For histopathological examination, tissue slices were stained with hematoxylin and eosin (H&E). For immunohistochemical testing, tissue slices were dewaxed, dehydrated, and microwaved for 20 min at 90 °C in a citrate buffer. A mouse anti-CA10 monoclonal antibody L8F12 (1:1000 dilution; gifted by Professor Xia’s laboratory, Xiamen University, Xiamen, China) was applied for 1 h at 37 °C. The peroxidase-conjugated secondary antibody was added for 30 min at room temperature, and then developed by diaminobenzidine tetrahydrochloride (DAB) chromogen solution (Fuzhou Maixin Biotechnology Development Co., Ltd., Fuzhou, China).

### Statistical analysis

The survival rates were evaluated by the Mantel-Cox log-rank test. The clinical scores and the average body weights were compared using Dunn’s multiple-comparison test. *P*-values (*P*) less than 0.05 were considered statistically significant.

## Results

### Construction of a full-length cDNA clone of CA10 via the in-fusion cloning strategy

The overall strategy to construct the infectious clone of CA10 is shown in Fig. 1a. The viral RNA was extracted and reverse transcribed using oligo (dT) primers. Two overlapped CA10 DNA fragment-1 and fragment-2 were amplified from the first strand cDNA. Fragment 1 contained 20 nucleotides of linearized pSVA vector at 5′ end and 1–3813 bp of the CA10 genome, while fragment-2 contained 20 nucleotides of linearized pSVA vector at 3′ end and 3794–7411 of the CA10 genome with poly(A) tails of 25 bases in length. The pSVA vector was linearized by PCR. The pSVA vector, CA10 DNA fragment-1and fragment-2 were then assembled to construct pSVA-CA10 plasmids using an In-Fusion Cloning Kit. Double (*Not I/Sal I*)and single (*Not I*) enzyme digestion identification showed that the length of CA10 genome, pSVA and pSVA-CA10 were all correct (Fig. 1b). The sequence of pSVA-CA10 was determined and there was no nucleotide mutation compared with the original CA10 strain sequence.

### Recovery and physicochemical feature of infectious CA10 from the cDNA clone

**Fig. 2:**
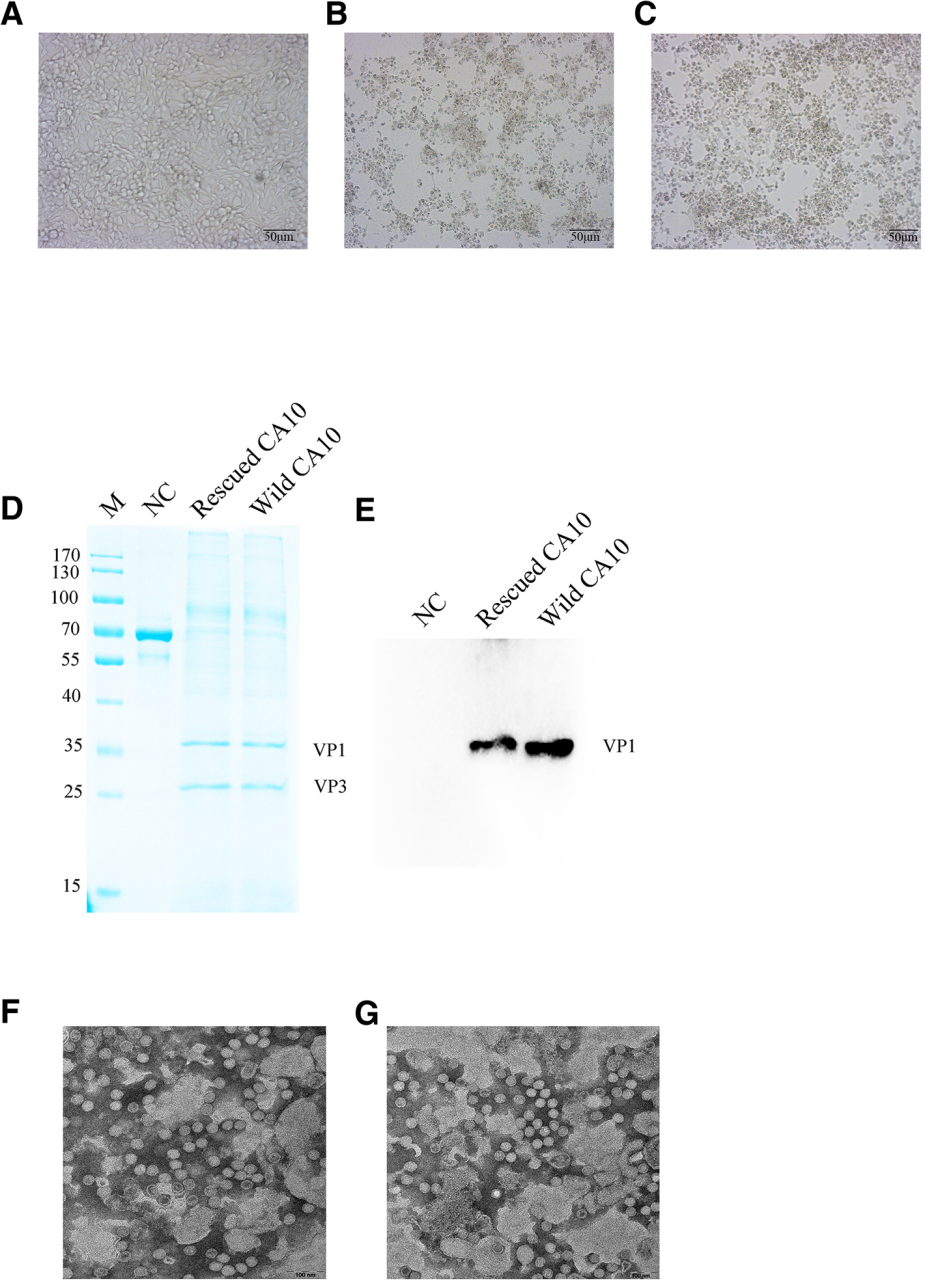
Identification of recovered CA10 from the cDNA clone. **a** Normal RD cells without infected virus. **b** Cytopathic effects displayed in RD cells were infected with the rescued viruses. **c** Cytopathic effects displayed in RD cells were infected with the wild CA10 viruses. **d** Identification of recovered CA10 and the wild CA10 by SDS-PAGE and (**e**) Western blotting. **f** and **g** are electron microscopic examinations of the purified recovered CA10 and wild CA10 particles. The scale bar is 100 nm

**Fig. 3:**
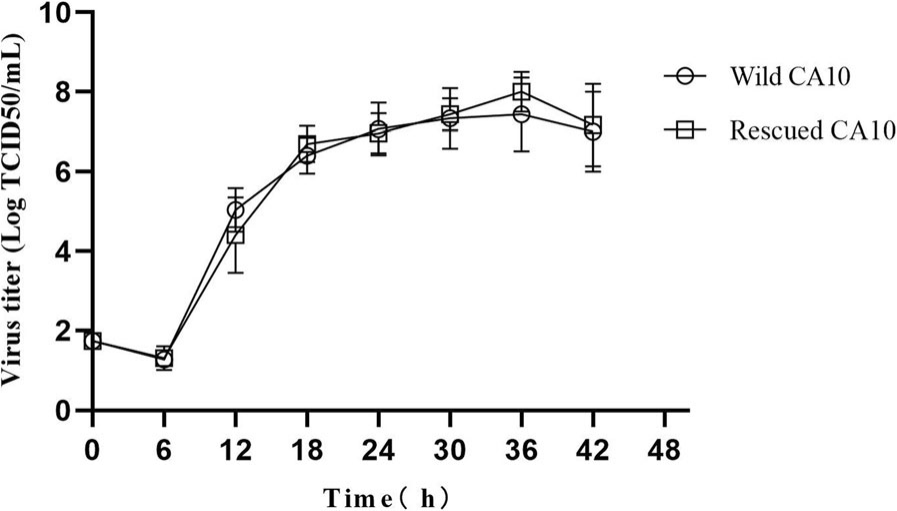
One-step growth curves of the wild and rescued CA10 viruses were analyzed by infecting RD cells with viruses at 50 TCID_50_ per well in 12-well plates. The supernatant was collected at 0, 6, 12, 18, 24, 30, 36 and 42 h after infection and titrated using a microtitration assay. At each time point, titer values are means of the three samples. Error bars represent the standard deviation from triplicate experiments

To recover CA10 virus, pSVA-CA10 and pLVX-Puro-T7-RNA polymerase DNA were co-transfected into 293 T cells. After 72 h, the supernatant was harvested and then used to infect RD cells. After another 72 h, cells were analyzed by a microscope, the cell supernatant was harvested and the titer of viruses was measured and cell precipitates were analyzed by biochemical assays. The recovered virus reached a maximum titer of10^8.125^TCID_50_/mL. As illustrated in Fig. 2a, the negative control (NC) RD cells appeared to grow normally, whereas the recovered CA10 (Fig. 2b) and the wild CA10 (Fig. 2c) infected cells displayed severe CPE. As shown in Fig. 2d, VP1 and VP3 proteins were detected in the supernatants of the RD cells infected with recovered CA10 or wild CA10 by SDS-PAGE. As shown in Fig. 2e, VP1 protein was detected by Western blotting in the supernatant of the RD cells infected with recovered CA10 or wild CA10. For morphological characterization of the rescued viruses, the viral particles were observed under TEM with magnification of 30000×. The rescued CA10 (Fig. 2f) or wild CA10 (Fig. 2g) particles were both about 30 nm in diameter, which were in agreement with the diameter of other enteroviruses of the *Piconaviridae* family. In addition, the growth rates between the wild and rescued CA10 viruses showed no significant difference, as shown in Fig. 3.

### CA10 infection in one-day-old mice resulted in severe illness and death

**Fig. 4:**
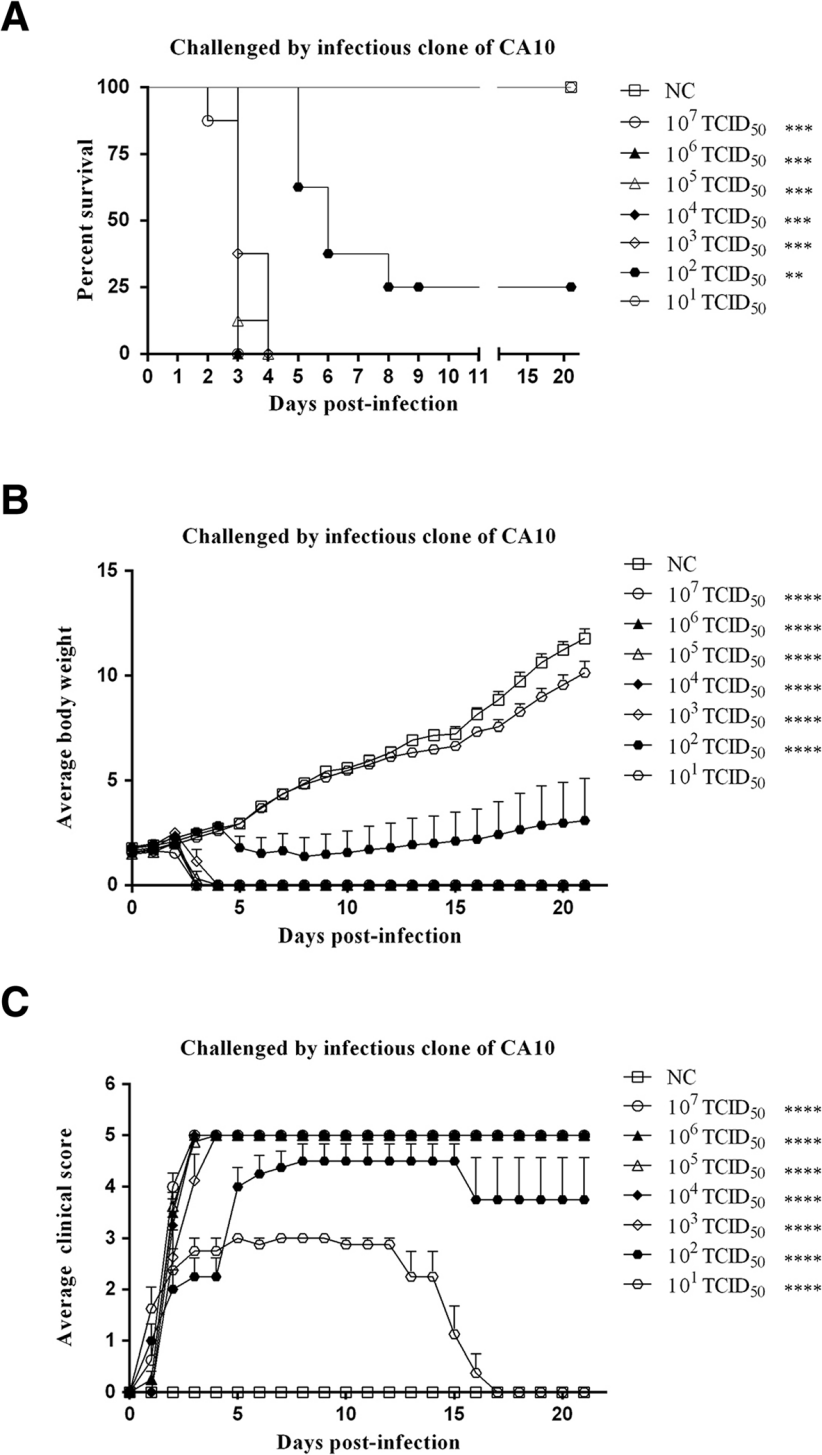
The virulence evaluation of the rescued CA10 viruses. Seven groups of one-day-old ICR mice were challenged with 10-fold serial dilution of recovered CA10 (10^7^ TCID50~10^1^ TCID50) via intracerebral routes. **a** Survival curve of the neonatal mice. **b** Average body weight of the neonatal mice. **c** Health scores of the neonatal mice. The survival rates were evaluated by the Mantel-Cox log-rank test. The clinical scores and the body weight were compared using Dunn’s multiple-comparison test. ****:*P* < 0.0001, ***:*P* < 0.001, **:*P* < 0.01

To assess the virulence of rescued CA10, 7 groups of one- day-old ICR mice were challenged with 10-fold serial dilution of recovered CA10 (10^7^–10^1^ TCID_50_) via intracerebral routes. Negative controls were alternatively challenged with PBS. The survival percentage, mean body weight, and average clinical scores were indicated in Fig. 4. The severity of clinical symptoms, from mild to severe, was scored as five grades. The mortality of the 10^7^~10^3^ TCID_50_ group was 100%, and the mean clinical scores were at grade 5 at 4 days post-infection. The survival rate of the 10^2^ TCID_50_ group was 25%, and the mean clinical scores were above grade 4 at 5 days post-infection, then decreased under grade 4 at 16 days post-infection. There was no death in 10^1^ TCID_50_ group, and the mean weight had no significant difference from negative control. The mean clinical scores of the 10^1^ TCID_50_ group were grade 3 at 4 days post-infection and decreased to 0 at 17 days post-infection. The Mantel-Cox log-rank test indicated that there was statistically significant difference in survival rates between NC group and 10^7^–10^2^ TCID_50_ groups. Dunn’s multiple-comparison test revealed that there was statistically significant difference in mean body weight between NC group and 10^7^–10^2^ TCID_50_ groups, and in mean clinical scores between NC group and 10^7^–10^1^ TCID_50_ groups. (****: *P*<0.0001; **: *P* < 0.01)..

### Pathological changes and viral distributions in infected mice after intracerebral challenge with a lethal dose of recovered CA10

**Fig. 5:**
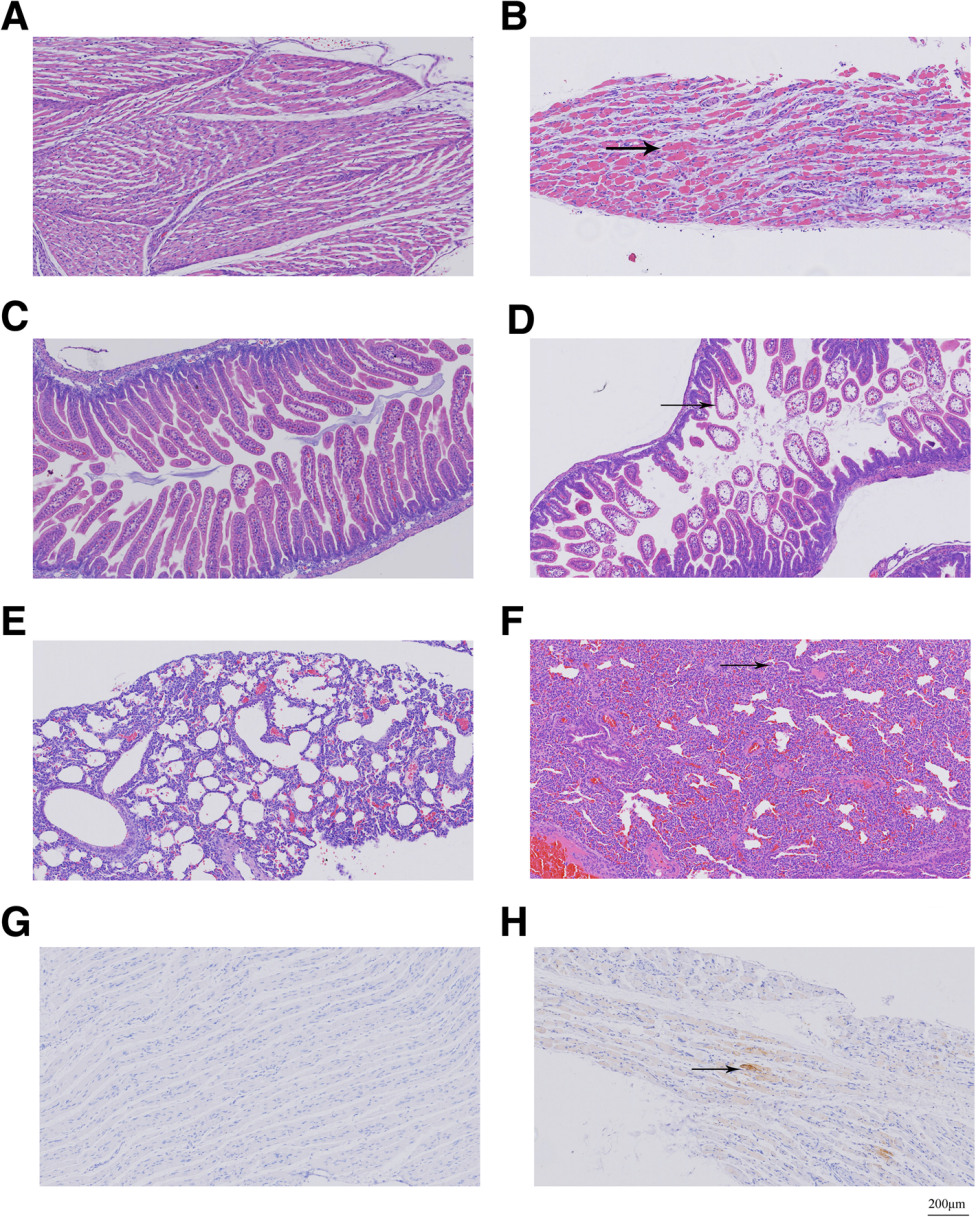
Histological (**a-f**) and immunohistochemical (**g-h**) examination of the infected neonatal mice in the moribund state. **a** The skeletal muscle of the mock-infected control mice. **b** The section of infected mice skeletal muscles exhibited severe necrotizing myositis (arrow). **c** The small intestine of the mock-infected control mice. **d** The section of infected mice small intestine exhibited intestinal villus interstitial edema and epithelial cell vacuolar degeneration. **e** The lung of the mock-infected control mice. **f** The section of infected mice lung exhibited severe alveolar shrinkage, vascular dilatation and congestion. **g** and **h** are based on immunohistochemical examination of the infected neonatal mice in the moribund state. **g** No viral antigen was detected in skeletal muscles of the mock control mice. **h** In contrast, distinct viral antigen was observed in skeletal muscles fibers of infected mice (arrow)

To investigate the pathological changes, multiple tissues of neonatal ICR mice infected with CVA10 in a moribund state were examined by H&E staining. The limb skeletal muscle of infected mice exhibited severe necrotizing myositis, and muscle fibers were irregularly scattered in fractures or disappeared (Fig. 5b). The small intestine of some infected mice showed intestinal villus interstitial edema and scattered epithelial cell vacuolar degeneration (Fig. 5d). Meanwhile, the lung of infected mice showed severe alveolar shrinkage, vascular dilatation and congestion, while no pulmonary fibrosis and inflammatory cell infiltration were found (Fig. 5f). In addition, no obvious pathological change observed in the brain, heart or liver and no viral antigen was detected in brain, heart, liver, intestine or lung (data were not shown). For determination of viral distribution, viral RNA in tissues was quantified by real-time PCR, and viral antigen was detected by immunohistochemical staining. The real-time PCR results showed that the amount of virus RNA was maximum in limb skeletal muscle (8.9 × 10^8^ copies/mL). The immunohistochemical staining indicated that CA10 antigen was only detected in limb skeletal muscles (Fig. 5h), which suggested the limb skeletal muscle as the most likely site for in vivo replication.

## Discussion

The development of infectious clone technology enables manipulation of RNA viruses at the molecular level and provides an effective method for the study of the structure and function of RNA virus genomes. In this study, we, for the first time, exhibited the construction of an infectious full-length CA10 cDNA clone from the P148/ZS/CHN/2012 strain, as well as recovery of CA10 viruses from the infectious clone. Our data demonstrated that co-transfection of pSVA-CA10 DNA with pLVX-Puro-T7 RNA polymerase DNA could produce infectious rescued CA10 viruses. The recovered CA10 was compared with the wild CA10 virus by CPE, SDS-PAGE, western blotting, TEM and growth rates. The results showed that the rescued virus and its parental strain shared similar physical and chemical characteristics, which proved that the CA10 infectious clone had been successfully constructed and could be used as a specific template for preservation of the CA10 strain.

In previous studies, infectious clones were successfully developed for a number of enteroviruses with different methods. EV71 or CA16 cDNA clones were established by assembling segmented cDNA fragments of the viral genome into a DNA vector via specific cleavage sites [15, 23, 24], and CA6 or Echo25 cDNA clones were constructed by utilizing In-Fusion Cloning to assemble the full length of viral genome and vector, which was difficult to operate. [16, 19]. In the present study, the CA10 genome was amplified into two close-sized segments, and the two DNA segments were connected to the linearized vector using the In-Fusion Cloning only in one step.

Furthermore, the virulence of the recovered CA10 virus in neonatal mice was evaluated, the organ or tissue lesions caused by the virus in vivo were analyzed by H&E staining, and the main tissue of the virus distribution in infected mice was investigated by immunohistochemistry and real-time PCR. The results disclosed that the mortality of the challenged mice was increased with the increase of virus concentrations, and mice in 10^7^ -10^3^ TCID_50_ dilution groups showed severe clinical signs, such as wasting, limb-shake weakness and hind-limb paralysis. The histopathological analysis showed that CA10 infection induced obvious tissue lesions, including scattered limb skeletal muscle fibers, intestinal villus interstitial edema and alveolar shrinkage, however, notable inflammatory symptoms were not detected in the majority of tissues. Additionally, in contrast to other types of enterovirus, EV71 and CA16 not only had a muscle tropism, but also could enter the brain and spinal cord [22, 25–27]; In the present research, we only detected viral antigens in limb skeletal muscle of the infected mice by immunohistochemical staining, and the virus copies in limb skeletal muscles were significantly higher than those in other tissues, which suggested that the limb skeletal muscle was the primary location of viral replication. Our results mentioned above were consistent with a previous research [28], which established a neonatal mouse model directly with a CVA10 clinical strain (CVA10-FJ-01), but not from an infectious clone. Thus, theCA10 infectious clone can be used for the preservation of specific virus strains, and it also can be used for establishment of mouse models, serving as a reference for human infection with the same virus.

## Conclusions

In conclusion, we for the first time successfully established an infectious cDNA clone of CA10. The CA10 virus recovered from this cDNA clone was genetically and biologically identical to its parental strain, and could induce mouse’s multiple tissue lesion and death after intracerebral infection, which thus could be used for establishment of a virus strain model. In brief, this study will facilitate the next researches related to the viral gene functions, pathogenesis, or vaccine development in the future.

## Data Availability

Not applicable.

## Abbreviations

CA10: Coxsackievirus A10
CA16: Coxsackievirus A16
CPE: Cytopathic effect
DMEM: Dulbecco’s Modified Eagle Medium
ECHO: Enteric cytopathic human orphan virus
EV71: Enterovirus A71
FBS: Fetal bovine serum
HE: Hematoxylin and eosin
HFMD: Hand, foot and mouth disease
ICR: Institute of Cancer Research
IHC: Immunohistochemical
PEG: Polyethylene glycol
RD: Rhabdomyosarcoma
SPF: Specific pathogen-free
TCID50: 50% tissue culture infectious dose
